# NIV through the helmet can be used as first-line intervention for early mild and moderate ARDS: an unproven idea thinking out of the box

**DOI:** 10.1186/s13054-019-2429-2

**Published:** 2019-06-14

**Authors:** Massimo Antonelli

**Affiliations:** Department of Anesthesiology, Intensive Care Medicine and Toxicology, Fondazione Policlinico Universitario A. Gemelli IRCCS-Università Cattolica del Sacro Cuore, Rome, Italy

**Keywords:** ARDS, Hypoxemic respiratory failure, Helmet pressure support

The lunatic, the lover, and the poet, are of imagination all compact.

Are you sure/That we are awake? It seems to me/That yet we sleep, we dream

William Shakespeare, A Midsummer Night’s Dream

Debating data have been published as regards the beneficial or deleterious effect of spontaneous breathing (SB) in comparison to controlled mechanical ventilation (CMV) during acute respiratory failure [1, 2].

Spontaneous breathing (SB) has been shown having several beneficial effects such as improving ventilation-perfusion matching and decreasing muscle atrophy and ventilator-induced lung injury (VILI) [3, 4].

There are experimental evidences that SB can also cause or worsen lung injury during mechanical ventilation [5, 6].

The implicated mechanisms include negative intra-thoracic and increased trans-alveolar pressure with a lack of control of tidal volume (VT), ventilation inhomogeneity and cyclic and static overinflation [7].

In animals with severe lung injury, SB could worsen lung injury. Muscle paralysis might be more protective by preventing injuriously high transpulmonary pressure and high driving pressure [8].

One of the most important determinants of the ventilator-induced lung injury is considered the transpulmonary pressure, that is, calculated as *P*_L_ = *P*_ao_ − *P*_pl_, where *P*_L_ is the difference between the pressure at the airway opening and the pleural or oesophageal pressure (used as a surrogate of the pleural pressure).

During SB, the airway pressure (*P*_aw_) is lower than during CMV, but this does not always translate into a lower pressure across the lung (i.e. a lower *P*_L_).

Only the transalveolar pressure, which equals the product of lung elastance and volume, is dissipated across the alveolus and is usually considered to cause VILI.

Instead of the absolute value of transpulmonary pressures, some investigators identify the lung stress with the variation of the transpulmonary pressure between end inspiration and end expiration, obtained during occlusion manoeuvres. All these manoeuvres are quite complicated to be performed while patients’ breathing spontaneously, especially under pressure support ventilation (PSV) and their validity, is put in question.

However, obtaining reliable physiological measurements in patients during noninvasive ventilation (NIV) or/and in patients spontaneously breathing without an endotracheal tube is extremely difficult, and the measurement cannot be reliably achieved through the conventional manoeuvres.

The only study that reports some interesting physiological measurements was the one published by L’Her et al. who showed that noninvasive pressure support of 10–15 cm H2O above a positive end-expiratory pressure (PEEP) of 5–10 cm H_2_O was the best combinations to reduce the inspiratory muscle effort, oesophageal pressure and dyspnoea and improve oxygenation [9]. In addition, experiments conducted on trained marathon runners in the sixties and more recently in endurance-trained individual put in evidence that the mechanism of spontaneous breathing-induced lung damage is not really understood. Indeed, these individuals during the exercise develop potentially injurious tidal volumes (TV) > 3 l, minute volumes (MV) (exceeding the 160 l/min) and transpulmonary pressures (ranging from − 40 cm H_2_O up to + 60 cm H_2_O) without developing any lung damage [10, 11].

Consequently, the question whether the noninvasive ventilation preserving the spontaneous breathing can be safely used for moderate and mild ARDS remains substantially unanswered.

Noninvasive positive pressure ventilation has been convincingly shown to be safe and effective as first-line treatment in patients with acute hypercapnic respiratory failure and acute cardiogenic pulmonary oedema [12–15]. Despite some data suggest that NIV may also avoid intubation in heterogeneous categories of patients with acute hypoxemic respiratory failure [16–22], its safety and efficacy in such a context is still debated, given the high failure rate and the possible detrimental effect on the clinical outcome [22–34].

As patients’ comfort is crucial for NIV success, over the last years, a great effort has been made to optimize NIV tolerability. Different interfaces are available for noninvasive ventilation [35]: in spite of face masks being more commonly used, helmet has been shown to improve patients’ comfort, allowing patients’ interaction, speech and feeding and not limiting cough. Nonetheless, skin necrosis, gastric distension or eye irritation are seldom observed during helmet NIV, while these may be consequences of long-term treatments with face masks [36, 37].

Moreover, differently from face masks, helmets permit longer-term treatments and allow the setting of higher levels of PEEP without causing air leaks or important patient-ventilator asynchrony; this aspect may be crucial when treating severely hypoxemic patients with acute respiratory failure and the acute respiratory distress syndrome (ARDS) [38]. Interestingly, higher PEEP during fully controlled mechanical ventilation in the early phase of the disease improves mortality in ARDS patients, and raising evidence indicates that it may exert beneficial effects also if spontaneous breathing is maintained [38, 39]. As a general rule, more severe patients (those with lower FRC and a higher shunt mechanism) are more recruitable and most benefit from higher PEEP that can be assured through the helmet during NIV with minimal leaks.

Helmet may allow NIV to fully exert its beneficial effects. In this sense, a recent randomized controlled trial comparing continuous NIV delivered with helmet or face-mask in patients with ARDS showed a lower intubation rate and a lower 90-day mortality in patients in the helmet group who, accordingly, underwent treatments with higher PEEP and lower FiO_2_ [40]. In this study, however, pressure support (PSV) delivered with NIV and low-flow-continuous positive airway pressure (CPAP) were indifferently used in patients randomized to the helmet group, despite their mechanisms of action, efficacy and potential harmful effects are profoundly different, especially given the high relevance of the driving pressure in such a context [41].

No study has ever clarified whether first-line treatment with helmet NIV as compared to other forms of oxygen support or invasive ventilation may yield a significant benefit to critically ill patients with respiratory failure.

The unproven idea that captured my imagination, needing a specific trial aimed to confirm our observational data, was using the noninvasive ventilation through the helmet as a tool for the early treatment of a mild and moderate form of ARDS.

A human being should follow the inspiration.
